# Integrity in Medical Colleges

**Published:** 1890-02

**Authors:** 


### Integrity in Medical Colleges.

It is a sign full of good omen that just now the attention of the members of the medical profession in this country is being called, in a number of differeng places, to the shame of dishonesty. For some years past there has been an apparent indifference in the profession to the questionable methods of some medical journals and medical schools, and, indeed, of certain medical practitioners. But now there seems to be a sort of awakening, and from various directions indications appear that it is only a temporary and seeming success which can be secured by ways which honorable men do not approve. It is well, we think, that responsible medical journals should, when occasion offers, express the sentiment of the best part—and by far the greater part—of the profession, so that those who might mistake its silence may understand clearly that the way to attain true success is to avoid not only the committal, but also the appearance of evil. Not long ago, one of our contemporaries pointed out the practical deception in the way in which a medical school printed the names of its graduates and matriculants, so that a false impression might be made as to their numbers. This is a fault which may not be very serious in itself, and one which admits of a plausible explanation; but it shows the natural inclination to swerve from the path of exact rectitude when there is some temptation to do so. Instances of like frailty might be cited from a number of medical schools in this country, but at this time a single case is enough to suggest the thought we wish to present, and to justify our pointing to the general sentiment of the profession, which, if properly appreciated, will no doubt prove sufficiently strong to correct a number of errors which now exist, so that these fountains of instruction may furnish also examples of general management and of personal character which their alumni may not fear to cite as ideals of what such institutions should do and be.—*Medical and Surgical Reporter.*

THE Norwegians are the longest-lived people in the world, there being a very slight mortality among children in that microbe-free land.

